# Transitions between body mass index categories, South Africa

**DOI:** 10.2471/BLT.20.255703

**Published:** 2020-09-28

**Authors:** Muchiri E Wandai, Jens Aagaard-Hansen, Samuel OM Manda, Shane A Norris

**Affiliations:** a SAMRC Developmental Pathways for Health Research Unit, University of the Witwatersrand, 27 St Andrew’s Road, Parktown, Johannesburg 2193, South Africa.; b Steno Diabetes Center Copenhagen, Gentofte, Denmark.; c Biostatistics Research Unit, South Africa Medical Research Council (SAMRC), Pretoria, South Africa.

## Abstract

**Objective:**

To profile the prevalence of the three body mass index (BMI) categories by sociodemographic characteristics, and to calculate the percentage transitioning (or not) from one BMI category to another, to inform South African health policy for the control of obesity and noncommunicable diseases.

**Methods:**

We used data from the National Income Dynamics Study, including sociodemographic characteristics and BMI measurements collected in 2008, 2010, 2012, 2014 and 2017. For each data collection wave and each population group, we calculated mean BMI and prevalence by category. We also calculated the percentage making an upwards transition (e.g. from overweight to obese), a downwards transition or remaining within a particular category. We used a multinomial logistic regression model to estimate transition likelihood.

**Findings:**

Between 2008 and 2017, mean BMI increased by 2.3 kg/m^2^. We calculated an increased prevalence of obesity from 19.7% (3686/18 679) to 23.6% (3412/14 463), with the largest increases in prevalence for those aged 19–24 years and those with at least high school education. The percentages of upwards transitions to overweight or obese categories increased sharply between the ages of 19 and 50 years. Once overweight or obese, the likelihood of transitioning to a normal BMI is low, particularly for women, those of higher age groups, and those with a higher income and a higher level of education.

**Conclusion:**

In the development of national strategies to control obesity and noncommunicable diseases, our results will allow limited public health resources to be focused on the relevant population groups.

## Résumé

**Objectif:**

Établir la prévalence des trois catégories d'indice de masse corporelle (IMC) en fonction des caractéristiques sociodémographiques, et calculer le pourcentage de transition (ou d'absence de transition) d'une catégorie d'IMC à l'autre, afin de renseigner les politiques de santé sud-africaines visant à lutter contre l'obésité et les maladies non transmissibles.

**Méthodes:**

Nous avons exploité les données provenant de l'Enquête nationale sur la dynamique des revenus (NIDS), notamment les caractéristiques sociodémographiques et les mesures de l'IMC récoltées en 2008, 2010, 2012, 2014 et 2017. Pour chaque vague de collecte de données et chaque groupe de population, nous avons calculé l'IMC moyen et la prévalence par catégorie. Nous avons également déterminé le pourcentage de passages vers la catégorie supérieure (en surpoids à obèse par exemple), de passages vers la catégorie inférieure et de maintien au sein d'une catégorie spécifique. Enfin, nous avons employé un modèle de régression logistique multinomiale pour estimer la probabilité de transition.

**Résultats:**

Entre 2008 et 2017, l'IMC moyen a augmenté de 2,3 kg/m^2^. Nous avons observé une prévalence accrue pour l'obésité, passée de 19,7% (3686/18 679) à 23,6% (3412/14 463). Cette hausse touche principalement les 19–24 ans et ceux possédant au moins un diplôme d'études secondaires. Le pourcentage de passage à la catégorie supérieure, vers le surpoids ou l'obésité, a fortement augmenté chez les personnes âgées de 19 à 50 ans. Une fois en surpoids ou obèse, la probabilité de repasser à un IMC normal est faible, surtout chez les femmes, les personnes plus âgées, ceux dont les revenus sont plus importants et dont le niveau d'éducation est plus élevé.

**Conclusion:**

Dans le cadre du développement de stratégies nationales destinées à lutter contre l'obésité et les maladies non transmissibles, nos résultats permettront d'affecter des ressources de santé publique limitées aux groupes de population les plus touchés.

## Resumen

**Objetivo:**

Describir la prevalencia de las tres categorías del índice de masa corporal (IMC) por características sociodemográficas y calcular el porcentaje que pasa (o no) de una categoría del IMC a otra con el fin de fundamentar la política sanitaria de Sudáfrica para controlar la obesidad y las enfermedades no transmisibles.

**Métodos:**

Se usaron los datos del Estudio nacional de la dinámica de los ingresos (NIDS, por sus siglas en inglés), incluidas las características sociodemográficas y las mediciones del IMC que se obtuvieron en 2008, 2010, 2012, 2014 y 2017. Se calculó el IMC medio y la prevalencia por categoría para cada fase de recopilación de datos y cada grupo de población. También se calculó el porcentaje que hace una transición ascendente (por ejemplo, del sobrepeso a la obesidad), una transición descendente o que permanece dentro de una categoría en particular. En este contexto, se aplicó un modelo de regresión logística polinomial para estimar la probabilidad de la transición.

**Resultados:**

Entre 2008 y 2017, el IMC medio aumentó en 2,3 kg/m². Se calculó un aumento de la prevalencia de la obesidad del 19,7 % (3686/18 679) al 23,6 % (3412/14 463), siendo los mayores aumentos de la prevalencia para las personas de 19 a 24 años de edad y las que habían cursado al menos estudios secundarios. Los porcentajes de transiciones ascendentes a las categorías de sobrepeso u obesidad aumentaron de manera considerable entre los 19 y los 50 años de edad. Cuando se tiene sobrepeso o se es obeso, la probabilidad de pasar a un IMC normal es baja, en especial en el caso de las mujeres, las de los grupos de edad avanzada y las que tienen mayores ingresos y un nivel de educación más alto.

**Conclusión:**

Los resultados obtenidos permitirán destinar los recursos limitados de la salud pública a los grupos de población correspondientes en la elaboración de estrategias nacionales para controlar la obesidad y las enfermedades no transmisibles.

## ملخص

الغرض تحديد مدى انتشار فئات مؤشر كتلة الجسم الثلاث (BMI) حسب الخصائص الاجتماعية السكانية، وحساب النسبة المئوية للتحول (أو عدم التحول) من فئة مؤشر كتلة جسم إلى أخرى، لإطلاع السياسة الصحية لجنوب إفريقيا لمكافحة السمنة والأمراض غير المعدية.

الطريقة استخدمنا بيانات من دراسة ديناميكيات الدخل القومي، بما في ذلك الخصائص الاجتماعية السكانية وقياسات مؤشر كتلة الجسم (BMI) التي تم جمعها في أعوام 2008 و2010 و2012 و2014 و2017. لكل موجة من مجموعات البيانات وكل مجموعة سكانية، قمنا بحساب متوسط ​​مؤشر كتلة الجسم والانتشار حسب الفئة. قمنا أيضًا بحساب النسبة المئوية التي تقوم بالتحول لأعلى (على سبيل المثال من زيادة الوزن إلى السمنة)، أو التحول لأسفل، أو البقاء ضمن فئة معينة. لقد استخدمنا نموذجًا للتحوف اللوجيستي متعدد الحدود لتقدير احتمالية التحول.

النتائج بين عامي 2008 و2017، زاد متوسط مؤشر كتلة الجسم بمعدل 2.3 كجم/متر ^2 ^. قمنا بحساب انتشار متزايد في السمنة من 19.7% (3686/18679) إلى 23.6% (3412/14463)، مع أكبر زيادات في الانتشار لمن تتراوح أعمارهم بين 19 و24 عامًا وأولئك الذين حصلوا على تعليم في المدرسة الثانوية على الأقل. زادت النسب المئوية للتحولات إلى أعلى، أو إلى فئات الوزن الزائد أو السمنة، بشكل حاد بين سني 19 و50 عامًا. بمجرد الوجود في فئة زيادة الوزن أو السمنة، يكون احتمال الانتقال إلى مؤشر كتلة الجسم العادي منخفضًا، خاصة بالنسبة للنساء، وكذلك ذوي الفئات العمرية الأعلى، وذوي الدخل المرتفع والمستوى التعليمي الأعلى.

الاستنتاج عند تطوير الاستراتيجيات الوطنية للسيطرة على السمنة والأمراض غير المعدية، سوف تسمح النتائج لدينا بتركيز موارد الصحة العامة المحدودة على المجموعات السكانية ذات الصلة.

## 摘要

**目的:**

通过社会人口统计学特征，分析三类身体质量指数 (BMI) 的患病率，并计算从 BMI 某个类别转换（或不转）为另一类的比例，从而为南非控制肥胖和非传染性疾病的卫生政策提供依据。

**方法:**

我们利用国民收入动态追踪调查研究数据，包括 2008 年、2010 年、2012 年、2014 年以及 2017 年收集的社会人口统计学特征和 BMI 测量结果。针对各数据收集曲线和各人口群体，我们计算出各类 BMI 平均值和患病率。还分别计算出向上转换（例如，从超重到肥胖）、向下转换或维持在特定类别的比例。我们使用多类别逻辑回归模型预估转换可能性。

**结果:**

2008 年至 2017 年间，BMI 平均值增长了 2.3 kg/m^2^。我们计算出，肥胖患病率从 19.7% (3686/18 679) 上升到 23.6% (3412/14 463)，其中 19-24 岁人群和具有中学以上学历的人群患病率增幅最大。19 至 50 岁间向上转换为超重或肥胖类别的比例急剧增加。一旦超重或肥胖，再转换为正常 BMI 的可能性较小，特别是女性、年龄较大的人群，以及收入较高和受教育程度较高的人群。

**结论:**

制定控制肥胖和非传染性疾病的国家策略时，我们的结果有助于将有限的公共卫生资源集中用于相关人群。

## Резюме

**Цель:**

Составить профиль распространенности трех категорий индекса массы тела (ИМТ) по социально-демографическим характеристикам и рассчитать процент перехода (или отсутствия такового) из одной категории ИМТ в другую, чтобы предоставить информацию для политики здравоохранения Южной Африки в целях борьбы с ожирением и неинфекционными заболеваниями.

**Методы:**

Авторы использовали данные Национального исследования динамики доходов, включая социально-демографические характеристики и измерения ИМТ, которые были собраны в 2008, 2010, 2012, 2014 и 2017 годах. Для каждого цикла сбора данных и каждой группы населения был рассчитан средний ИМТ с разбивкой показателей по категориям. Авторы также рассчитали процент восходящих переходов (например, из категории избыточного веса в категорию ожирения), нисходящих переходов и процент постоянно пребывающих в определенной ИМТ-категории. Для оценки вероятности перехода между категориями использовалась модель мультиномиальной логистической регрессии.

**Результаты:**

В период с 2008 по 2017 год средний показатель ИМТ увеличился на 2,3 кг/м^2^. Авторы рассчитали рост распространенности ожирения с 19,7% (3686/18 679) до 23,6% (3412/14 463), при этом наибольший рост распространенности ожирения наблюдается среди лиц в возрасте 19–24 лет и лиц, имеющих уровень образования не ниже среднего. Процент восходящих переходов в категории с избыточным весом или ожирением резко увеличился в возрастной категории от 19 до 50 лет. После попадания в категорию избыточного веса или ожирения вероятность перехода к нормальному ИМТ является низкой, особенно для женщин, лиц старших возрастных групп и лиц с более высоким доходом и более высоким уровнем образования.

**Вывод:**

При разработке национальных стратегий борьбы с ожирением и неинфекционными заболеваниями полученные результаты позволят сосредоточить внимание ограниченных ресурсов общественного здравоохранения на соответствующих группах населения.****

## Introduction

The mean body mass index (BMI) of the African population is increasing,^1^ resulting in a steady rise of the prevalence of people being overweight or obese across Africa, with the southern part of Africa being most affected.^1^^,^^2^ In 2016, the prevalence of the population aged ≥ 15 years being overweight or obese in South Africa was 68% for women and 31% for men.^3^

Global efforts to combat obesity include the World Health Organization (WHO) *Global strategy on diet, physical activity and health*,^4^ the *Global action plan for the prevention and control of noncommunicable diseases 2013–2020*^5^ and the United Nations (UN) *High-level meetings of the General Assembly on prevention and control of non-communicable diseases*.^6^^,^^7^ The *Global action plan* proposes the promotion of healthy diets by Member States to halt the rise in the prevalence of school children, adolescents and adults being overweight or obese. Similarly, the 2011 Sixty-sixth session of the UN Political Declaration of the High-level Meeting of the General Assembly on the Prevention and Control of Non-communicable Diseases committed to strengthening national policies and health systems by promoting multisectoral and multistakeholder engagement to reverse, stop and decrease the rising trends of obesity in child, youth and adult populations.^6^ In line with global strategies and policies, the South African Department of Health developed the *Strategic plan for the prevention and control of non-communicable diseases 2013–2017*^8^ and the *Strategy for the prevention and control of obesity in South Africa 2015–2020*;^9^ the targets of these strategic plans were to reduce obesity prevalence by 3% by 2017 and by 10% by 2020 in all age groups. These two strategic plans are aligned with the agenda of the country’s 2030 *National development plan* for the promotion of healthy diets and physical activity at schools, workplaces and in the general community.^10^

Promotion and support through research is an essential component of global and national strategies for prevention and control of obesity and noncommunicable diseases.^4^^,^^5^^,^^8^^,^^9^ We anticipate that a better understanding of transitions between the BMI categories – normal, overweight and obese (Table 1)^11^ – will allow the improvement of interventions to reduce the prevalence of obesity. Our objectives are: (i) to profile the prevalence of the three BMI categories within a study population according to various sociodemographic characteristics, and to estimate the percentage of these population groups that underwent transitions (or not) between BMI categories; (ii) to identify the factors associated with transitions between BMI categories; and (iii) to discuss the key public health implications of our findings for national obesity control strategies.

**Table 1:** Body mass index categories for children and adults, according to WHO^11^

BMI category	*z*-BMI of children (≤ 18 years)	BMI of adults (> 19 years) (kg/m^2^)
Normal	> –2SD to ≤ 1SD	≥ 18.5 to < 25
Overweight	> 1SD to ≤ 2SD	≥ 25 to < 30
Obese	> 2SD	≥ 30

BMI: body mass index; SD: standard deviation of mean BMI; WHO: World Health Organization; *z*-BMI: *z* score for BMI = (BMI – mean BMI)/SD.

## Methods

### Study population

The National Income Dynamics Study, first conducted in 2008, is a nationally representative panel study that collects information on a wide variety of social, demographic, economic and health characteristics of the civilian non-institutionalized population.^12^^,^^13^ We used data from the five completed waves of the panel survey (the subsequent four waves were conducted in 2010, 2012, 2014 and 2017) from participants for which anthropometric measurements had also been recorded. In the first wave in 2008, the survey recorded weight and height measurements for the calculation of BMI for 21 002 individuals; 2323 (11.1%) were immediately lost to follow-up. Of the remaining 2008 sample of 18 679 individuals for which at least a second BMI calculation was recorded, 13 298 (71.2%), 15 331 (82.1%), 15 623 (83.6%) and 14 463 (77.4%) are represented in the 2010, 2012, 2014 and 2017 waves, respectively. 

### Study variables

Our main study variable of interest is whether transition occurred from one BMI category to another during a particular period in time. This derived variable has seven possible outcomes: two downwards transitions (either from obese to overweight or from overweight to normal); two upwards transitions (either from normal to overweight or from overweight to obese); and three no-transition outcomes, when a person’s BMI category does not change from either normal, overweight or obese between two waves of the survey. If a population is experiencing a higher number of upwards than downwards transitions, the prevalence of adverse conditions will increase.

Our independent time-invariant variables were sex and race, and baseline time-variant variables were age, whether urban or rural residence, education level, equivalized income level and frequency of physical exercise. To account for the economies of scale in household consumption, we used the equivalization method of the Organisation for Economic Co-operation and Development of dividing household income by the square root of the number of people within the household.^14^

### Statistical analysis

For each BMI category and for each of the five waves of data collection, we calculated both average BMI with 95% confidence intervals and prevalence. To visualize how prevalence varies with age, we calculated the prevalence of all three BMI categories for all ages and averaged over all five data collection waves (2008–2017). We also calculated the percentage within each population subgroup either transitioning to an upwards or downwards BMI category or remaining within the same category during the four periods between subsequent waves (i.e. ending in 2010, 2012, 2014 and 2017). We used a multinomial logistic regression to model the probability of transitioning (or not) from one BMI category to another, relative to the probability of remaining within the normal category. We performed all statistical analyses using Stata SE Version 15.0 (StataCorp, College Station, United States of America).

## Results

### Average BMI and prevalence

Between 2008 and 2017, mean BMI increased by 2.3 kg/m^2^, from 23.1 to 25.4 kg/m^2^. We observed that the age group 7–13 years experienced the highest increase (by 4.7 kg/m^2^), followed by the age groups 14–18 years (3.3 kg/m^2^) and 19–24 years (3.2 kg/m^2^; Table 2; available at: http://www.who.int/bulletin/volumes/98/12/20-255703). We note that the groups that demonstrated at least an average increase in mean BMI (i.e. ≥ 2.3 kg/m^2^) include women, Africans and Caucasians, rural dwellers, those with some education and those whose level of physical exercise was unknown. When examining the data by income, those with the lowest income demonstrated the largest increase in mean BMI (2.5 kg/m^2^). We also observed that women, those aged ≥ 25 years, Caucasians, urban dwellers, those with no formal schooling, those with a high school education or more, those within the highest-income tertile and those exercising < 3 times per week had an average BMI of > 25.0 kg/m^2^ for most, if not all, of the study period, contributing to the high prevalence of people being overweight and obese (Table 2).

**Table 2:** Mean body mass index and percentage of study population within each body mass index category by sociodemographic characteristics and data collection wave, South Africa, 2008–2017

Sociodemographic property/data collection wave	*n*	Mean BMI, kg/m^2^ (95% CI)		No. (%) within each category		
				Normal	Overweight	Obese
**Sex**						
Female						
2008	10 687	24.8 (24.5–25.1)		5 444 (50.9)	2 374 (22.2)	2 869 (26.8)
2010	7 887	26.3 (25.9–26.6)		3 504 (44.4)	1 881 (23.8)	2 502 (31.7)
2012	8 936	26.4 (26.1–26.7)		4 033 (45.1)	2 270 (25.4)	2 633 (29.5)
2014	9 040	27.3 (27.0–27.7)		3 853 (42.6)	2 154 (23.8)	3 033 (33.6)
2017	8 461	27.9 (27.5–28.2)		3 473 (41.0)	2 057 (24.3)	2 931 (34.6)
Male						
2008	7 992	21.1 (20.8–21.3)		5 885 (73.6)	1 290 (16.1)	817 (10.2)
2010	5 411	22.2 (21.9–22.6)		3 737 (69.1)	1 054 (19.5)	620 (11.5)
2012	6 395	22.4 (22.0–22.7)		4 539 (71.0)	1 196 (18.7)	660 (10.3)
2014	6 583	22.4 (22.1–22.7)		4 985 (75.7)	1 036 (15.7)	562 (8.5)
2017	6 002	22.5 (22.2–22.7)		4 552 (75.8)	969 (16.1)	481 (8.0)
**Baseline age (years)**						
0–6						
2008	2 770	16.8 (16.6–17.0)		1 792 (64.7)	544 (19.6)	434 (15.7)
2010	1 627	16.7 (16.4–16.9)		1 106 (68.0)	275 (16.9)	246 (15.1)
2012	2 468	17.0 (16.8–17.3)		1 780 (72.1)	387 (15.7)	301 (12.2)
2014	2 571	17.9 (17.6–18.1)		2 090 (81.3)	321 (12.5)	160 (6.2)
2017	2 479	19.2 (18.9–19.4)		2 029 (81.8)	314 (12.7)	136 (5.5)
7–13						
2008	3 458	17.8 (17.4–18.1)		2 781 (80.4)	378 (10.9)	299 (8.6)
2010	2 327	19.8 (19.4–20.2)		1 733 (74.5)	359 (15.4)	235 (10.1)
2012	2 973	20.7 (20.5–21.0)		2 306 (77.6)	485 (16.3)	182 (6.1)
2014	3 026	21.8 (21.5–22.0)		2 375 (78.5)	459 (15.2)	192 (6.3)
2017	2 807	22.5 (22.2–22.8)		2 128 (75.8)	442 (15.7)	237 (8.4)
14–18						
2008	2 384	21.5 (21.2–21.8)		1 899 (79.7)	329 (13.8)	156 (6.5)
2010	1 761	23.1 (22.7–23.4)		1 230 (69.8)	358 (20.3)	173 (9.8)
2012	1 803	23.7 (23.3–24.0)		1 254 (69.6)	368 (20.4)	181 (10.0)
2014	1 953	24.2 (23.9–24.5)		1 253 (64.2)	387 (19.8)	313 (16.0)
2017	1 803	24.8 (24.4–25.3)		1 070 (59.3)	392 (21.7)	341 (18.9)
19–24						
2008	2 079	23.5 (23.2–23.9)		1 445 (69.5)	407 (19.6)	227 (10.9)
2010	1 455	24.6 (24.1–25.1)		871 (59.9)	334 (23.0)	250 (17.2)
2012	1 563	25.5 (24.9–26.1)		847 (54.2)	395 (25.3)	321 (20.5)
2014	1 703	26.2 (25.7–26.7)		838 (49.2)	431 (25.3)	434 (25.5)
2017	1 581	26.7 (26.1–27.2)		718 (45.4)	414 (26.2)	449 (28.4)
25–34						
2008	2 199	25.8 (25.5–26.2)		1 184 (53.8)	536 (24.4)	479 (21.8)
2010	1 597	26.4 (25.9–26.9)		721 (45.1)	430 (26.9)	446 (27.9)
2012	1 721	26.9 (26.5–27.4)		741 (43.1)	493 (28.6)	487 (28.3)
2014	1 831	27.6 (27.1–28.0)		749 (40.9)	458 (25.0)	624 (34.1)
2017	1 705	27.4 (26.9–27.9)		689 (40.4)	425 (24.9)	591 (34.7)
35–44						
2008	1 966	27.7 (27.3–28.2)		790 (40.2)	495 (25.2)	681 (34.6)
2010	1 514	28.4 (27.8–29.0)		539 (35.6)	387 (25.6)	588 (38.8)
2012	1 634	28.6 (28.0–29.1)		562 (34.4)	425 (26.0)	647 (39.6)
2014	1 647	28.8 (28.2–29.4)		560 (34.0)	376 (22.8)	711 (43.2)
2017	1 556	28.7 (28.1–29.2)		507 (32.6)	379 (24.4)	670 (43.1)
45–54						
2008	1 687	28.8 (28.2–29.3)		630 (37.3)	400 (23.7)	657 (38.9)
2010	1 305	29.3 (28.6–29.9)		424 (32.5)	330 (25.3)	551 (42.2)
2012	1 415	29.4 (28.8–30.0)		458 (32.4)	378 (26.7)	579 (40.9)
2014	1 370	29.6 (28.9–30.2)		425 (31.0)	343 (25.0)	602 (43.9)
2017	1 260	29.5 (28.9–30.2)		403 (32.0)	320 (25.4)	537 (42.6)
> 55						
2008	2 136	28.4 (27.9–28.9)		808 (37.8)	575 (26.9)	753 (35.3)
2010	1 712	28.4 (27.9–28.9)		617 (36.0)	462 (27.0)	633 (37.0)
2012	1 754	28.0 (27.6–28.4)		624 (35.6)	535 (30.5)	595 (33.9)
2014	1 522	28.1 (27.5–28.7)		548 (36.0)	415 (27.3)	559 (36.7)
2017	1 272	28.0 (27.4–28.6)		481 (37.8)	340 (26.7)	451 (35.5)
**Race**						
African						
2008	15 621	22.9 (22.6–23.1)		9 592 (61.4)	3 049 (19.5)	2 980 (19.1)
2010	11 536	24.3 (24.0–24.6)		6 262 (54.3)	2 569 (22.3)	2 705 (23.4)
2012	12 941	24.2 (24.0–24.5)		7 312 (56.5)	2 937 (22.7)	2 692 (20.8)
2014	13 228	24.8 (24.5–25.0)		7 581 (57.3)	2 714 (20.5)	2 933 (22.2)
2017	12 317	25.3 (25.0–25.5)		6 945 (56.4)	2 557 (20.8)	2 815 (22.9)
Mixed ancestry						
2008	2 360	24.0 (23.3–24.6)		1 441 (61.1)	405 (17.2)	514 (21.8)
2010	1 400	24.4 (23.6–25.2)		821 (58.6)	254 (18.1)	325 (23.2)
2012	1 887	24.9 (24.3–25.4)		1 091 (57.8)	356 (18.9)	440 (23.3)
2014	1 923	25.4 (24.9–26.0)		1 087 (56.5)	332 (17.3)	504 (26.2)
2017	1 770	25.8 (25.3–26.3)		949 (53.6)	348 (19.7)	473 (26.7)
Asian						
2008	199	23.4 (22.9–23.9)		116 (58.3)	46 (23.1)	37 (18.6)
2010	128	23.8 (22.9–24.7)		72 (56.3)	31 (24.2)	25 (19.5)
2012	141	25.5 (25.0–26.0)		66 (46.8)	45 (31.9)	30 (21.3)
2014	152	24.4 (23.5–25.2)		77 (50.7)	42 (27.6)	33 (21.7)
2017	145	24.0 (22.9–25.1)		63 (43.4)	49 (33.8)	33 (22.8)
Caucasian						
2008	499	25.7 (24.6–26.8)		180 (36.1)	164 (32.9)	155 (31.1)
2010	234	26.9 (25.1–28.7)		86 (36.8)	81 (34.6)	67 (28.6)
2012	362	27.8 (26.8–28.8)		103 (28.5)	128 (35.4)	131 (36.2)
2014	320	28.2 (26.8–29.6)		93 (29.1)	102 (31.9)	125 (39.1)
2017	231	28.3 (26.8–29.7)		68 (29.4)	72 (31.2)	91 (39.4)
**Residence**						
Rural						
2008	10 849	22.2 (21.9–22.5)		6 916 (63.7)	2 060 (19.0)	1 873 (17.3)
2010	7 937	23.8 (23.5–24.1)		4 374 (55.1)	1 791 (22.6)	1 772 (22.3)
2012	8 420	23.6 (23.3–23.9)		4 912 (58.3)	1 916 (22.8)	1 592 (18.9)
2014	8 152	24.2 (23.9–24.4)		4 825 (59.2)	1 646 (20.2)	1 681 (20.6)
2017	7 501	24.7 (24.4–25.0)		4 369 (58.2)	1 546 (20.6)	1 586 (21.1)
Urban						
2008	7 830	24.0 (23.7–24.3)		4 413 (56.4)	1 604 (20.5)	1 813 (23.2)
2010	5 361	25.1 (24.6–25.5)		2 867 (53.5)	1 144 (21.3)	1 350 (25.2)
2012	6 911	25.3 (24.9–25.7)		3 660 (53.0)	1 550 (22.4)	1 701 (24.6)
2014	7 471	25.7 (25.4–26.1)		4 013 (53.7)	1 544 (20.7)	1 914 (25.6)
2017	6 962	25.8 (25.5–26.2)		3 656 (52.5)	1 480 (21.3)	1 826 (26.2)
**Education**						
None						
2008	1 761	25.2 (24.8–25.6)		921 (52.3)	382 (21.7)	458 (26.0)
2010	1 391	26.3 (25.8–26.7)		638 (45.9)	324 (23.3)	429 (30.8)
2012	1 437	25.8 (25.4–26.1)		687 (47.8)	384 (26.7)	366 (25.5)
2014	1 345	25.9 (25.5–26.2)		672 (50.0)	316 (23.5)	357 (26.5)
2017	1 168	26.1 (25.7–26.5)		583 (49.9)	274 (23.5)	311 (26.6)
Pre-school						
2008	2 603	16.8 (16.6–16.9)		1 666 (64.0)	522 (20.1)	415 (15.9)
2010	1 508	16.7 (16.5–16.9)		1 020 (67.6)	251 (16.6)	237 (15.7)
2012	2 324	16.9 (16.8–17.1)		1 677 (72.2)	358 (15.4)	289 (12.4)
2014	2 418	17.5 (17.4–17.7)		1 969 (81.4)	295 (12.2)	154 (6.4)
2017	2 338	18.9 (18.7–19.0)		1 919 (82.1)	290 (12.4)	129 (5.5)
Primary school^a^						
2008	11 504	23.0 (22.9–23.2)		7 356 (63.9)	2 043 (17.8)	2 105 (18.3)
2010	8 441	24.6 (24.5–24.7)		4 760 (56.4)	1 823 (21.6)	1 858 (22.0)
2012	9 335	24.8 (24.7–24.9)		5 374 (57.6)	2 057 (22.0)	1 904 (20.4)
2014	9 573	25.4 (25.3–25.6)		5 416 (56.6)	1 953 (20.4)	2 204 (23.0)
2017	8 864	25.9 (25.7–26.0)		4 848 (54.7)	1 884 (21.3)	2 132 (24.1)
High school and above						
2008	2 811	26.3 (26.1–26.6)		1 386 (49.3)	717 (25.5)	708 (25.2)
2010	1 958	27.4 (27.1–27.6)		823 (42.0)	537 (27.4)	598 (30.5)
2012	2 235	27.9 (27.6–28.2)		834 (37.3)	667 (29.8)	734 (32.8)
2014	2 287	28.6 (28.3–28.9)		781 (34.1)	626 (27.4)	880 (38.5)
2017	2 093	29.0 (28.7–29.3)		675 (32.3)	578 (27.6)	840 (40.1)
**Income level**						
Low						
2008	11 220	22.1 (21.9–22.4)		7 236 (64.5)	2 055 (18.3)	1 929 (17.2)
2010	8 162	23.7 (23.6–23.9)		4 625 (56.7)	1 771 (21.7)	1 766 (21.6)
2012	9 264	23.4 (23.3–23.6)		5 548 (59.9)	2 019 (21.8)	1 697 (18.3)
2014	9 582	24.0 (23.9–24.1)		5 861 (61.2)	1 858 (19.4)	1 863 (19.4)
2017	8 974	24.6 (24.4–24.7)		5 371 (59.9)	1 770 (19.7)	1 833 (20.4)
Middle						
2008	4 564	23.5 (23.1–23.9)		2 738 (60.0)	868 (19.0)	958 (21.0)
2010	3 243	24.6 (24.4–24.9)		1 777 (54.8)	683 (21.1)	783 (24.1)
2012	3 780	24.6 (24.3–24.8)		2 094 (55.4)	809 (21.4)	877 (23.2)
2014	3 776	25.1 (24.9–25.4)		2 060 (54.6)	757 (20.0)	959 (25.4)
2017	3 501	25.5 (25.3–25.8)		1 866 (53.3)	745 (21.3)	890 (25.4)
High						
2008	2 895	25.0 (24.6–25.5)		1 355 (46.8)	741 (25.6)	799 (27.6)
2010	1 893	26.1 (25.7–26.4)		839 (44.3)	481 (25.4)	573 (30.3)
2012	2 287	26.5 (26.2–26.7)		930 (40.7)	638 (27.9)	719 (31.4)
2014	2 265	27.0 (26.7–27.3)		917 (40.5)	575 (25.4)	773 (34.1)
2017	1 988	27.5 (27.2–27.8)		788 (39.6)	511 (25.7)	689 (34.7)
**Physical exercise**						
< 3 times per week						
2008	10 452	26.3 (26.0–26.5)		5 342 (51.1)	2 404 (23.0)	2 706 (25.9)
2010	7 950	27.2 (27.0–27.3)		3 521 (44.3)	2 002 (25.2)	2 427 (30.5)
2012	8 315	27.5 (27.3–27.6)		3 503 (42.1)	2 239 (26.9)	2 573 (30.9)
2014	8 444	27.8 (27.7–28.0)		3 440 (40.7)	2 068 (24.5)	2 936 (34.8)
2017	7 742	28.1 (27.9–28.3)		3 056 (39.5)	1 935 (25.0)	2 751 (35.5)
≥ 3 times per week						
2008	1 460	24.0 (23.5–24.6)		981 (67.2)	274 (18.8)	205 (14.0)
2010	985	24.7 (24.3–25.0)		591 (60.0)	228 (23.1)	166 (16.9)
2012	1 135	25.2 (24.9–25.5)		646 (56.9)	288 (25.4)	201 (17.7)
2014	1 143	25.6 (25.2–25.9)		623 (54.5)	266 (23.3)	254 (22.2)
2017	1 040	25.8 (25.4–26.1)		550 (52.9)	255 (24.5)	235 (22.6)
Unknown						
2008	6 767	17.6 (17.4–17.8)		5 006 (74.0)	986 (14.6)	775 (11.5)
2010	4 363	19.0 (18.8–19.1)		3 129 (71.7)	705 (16.2)	529 (12.1)
2012	5 881	19.3 (19.1–19.4)		4 423 (75.2)	939 (16.0)	519 (8.8)
2014	6 036	20.2 (20.0–20.3)		4 775 (79.1)	856 (14.2)	405 (6.7)
2017	5 681	21.2 (21.0–21.3)		4 419 (77.8)	836 (14.7)	426 (7.5)
**Total**						
2008	18 679	23.1 (22.9–23.4)		11 329 (60.7)	3 664 (19.6)	3 686 (19.7)
2010	13 298	24.5 (24.2–24.7)		7 241 (54.5)	2 935 (22.1)	3 122 (23.5)
2012	15 331	24.6 (24.3–24.8)		8 572 (55.9)	3 466 (22.6)	3 293 (21.5)
2014	15 623	25.1 (24.8–25.3)		8 838 (56.6)	3 190 (20.4)	3 595 (23.0)
2017	14 463	25.4 (25.1–25.6)		8 025 (55.5)	3 026 (20.9)	3 412 (23.6)

BMI: body mass index; CI: confidence interval.

^a^ Includes those who may have started high school but dropped out before completing.

**Fig. 1:**
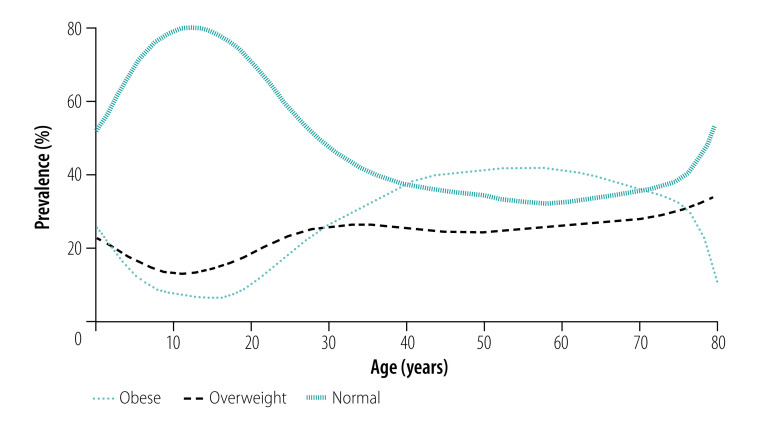
Prevalence of body mass index categories by age, South Africa

Note: Data are averaged over the five waves (2008, 2010, 2012, 2014 and 2017) of the National Income Dynamics Study data collection.

Our data show an increase in the prevalence of obesity from 19.7% (3686/18 679) in 2008 to 23.6% (3412/14 463) in 2017. In terms of age group, we observed the highest increase in the prevalence of obesity over this period in those aged 19–24 years from 10.9% (227/2079) to 28.4% (449/1581). In terms of education level, those with the most education (high school and above) demonstrated the largest increase in the prevalence of obesity from 25.2% (708/2811) to 40.1% (840/2093). The prevalence of people being overweight and obese was lowest for those aged around 11–18 years (Fig. 1). The prevalence of obesity increases steeply after this age up to around 50 years, when it reaches a plateau (Fig. 1). Between the ages of 40 and 70 years, the prevalence of obesity is higher than that of normal BMI.

### Transitions between categories 

We provide percentages of the study population groups who transitioned (or not) from one BMI category to another during the four periods ending 2010, 2012, 2014 and 2017 in Table 3 (available at: http://www.who.int/bulletin/volumes/98/12/20-255703). As the cohort for each risk factor aged throughout the study period, the percentages transitioning generally decreased as the percentages remaining within a particular BMI category increased. The exceptions to this general observation, that is, the groups that demonstrated decreasing percentages of retaining a normal BMI, included those of age 14–18 years (from 61.7%; 1086/1761 to 54.6%; 984/1803) and 19–24 years (from 51.6%; 751/1455 to 41.0%; 648/1581), Asians (from 51.6%; 66/128 to 38.6%; 56/145), individuals with at least a high school education (from 34.3%; 671/1958 to 28.1%; 589/2093) and those who reported physical exercise ≥ 3 times per week (from 53.0%; 522/985 to 48.6%; 505/1040). Within all these groups, our data show that the decreasing percentages of those retaining a normal BMI was accompanied by upwards transition percentages that were much higher than downwards transition percentages. For example, for the age group 19–24 years, the percentage transitioning upwards by 2010 (22.4%; (187+139)/1455) was more than double that of those transitioning downwards (10.1%; (100+47)/1455). The percentage of those retaining a normal BMI remained relatively constant throughout the study period for women (~35%) and for those aged > 35 years (~28%). At the end of every period, the percentage of those who retained a normal BMI was higher for those who exercised ≥ 3 times per week (e.g. for the period ending 2010, 53.0%; 522/985) compared with those who exercised < 3 times per week (e.g. for the period ending 2010, 36.1%; 2868/7950).

**Table 3:** Number and percentage of study population transitioning from one body mass index category to another (or not) by sociodemographic characteristics and year of end of transition period, South Africa, 2010–2017

Sociodemographic characteristic/end of transition period	*n*	No. (%)								
		Transitioned upwards			Transitioned downwards			Unchanged		
		Normal to overweight	Overweight to obese		Overweight to normal	Obese to overweight		Normal	Overweight	Obese
**Sex**										
Female										
2010	7 887	750 (9.5)	845 (10.7)		526 (6.7)	575 (7.3)		2752 (34.9)	782 (9.9)	1657 (21.0)
2012	8 936	844 (9.4)	790 (8.8)		602 (6.7)	826 (9.2)		3097 (34.7)	934 (10.5)	1843 (20.6)
2014	9 040	775 (8.6)	876 (9.7)		546 (6.0)	470 (5.2)		3142 (34.8)	1074 (11.9)	2157 (23.9)
2017	8 461	521 (6.2)	481 (5.7)		382 (4.5)	364 (4.3)		3022 (35.7)	1241 (14.7)	2450 (29.0)
Male										
2010	5 411	563 (10.4)	390 (7.2)		361 (6.7)	285 (5.3)		3201 (59.2)	381 (7.0)	230 (4.3)
2012	6 395	559 (8.7)	362 (5.7)		531 (8.3)	441 (6.9)		3725 (58.2)	479 (7.5)	298 (4.7)
2014	6 583	388 (5.9)	227 (3.4)		530 (8.1)	359 (5.5)		4224 (64.2)	520 (7.9)	335 (5.1)
2017	6 002	288 (4.8)	142 (2.4)		269 (4.5)	164 (2.7)		4232 (70.5)	568 (9.5)	339 (5.6)
**Baseline age (years)**										
0–6										
2010	1 627	128 (7.9)	168 (10.3)		189 (11.6)	177 (10.9)		806 (49.5)	81 (5.0)	78 (4.8)
2012	2 468	165 (6.7)	223 (9.0)		254 (10.3)	309 (12.5)		1333 (54.0)	106 (4.3)	78 (3.2)
2014	2 571	142 (5.5)	92 (3.6)		243 (9.5)	247 (9.6)		1668 (64.9)	111 (4.3)	68 (2.6)
2017	2 479	130 (5.2)	62 (2.5)		116 (4.7)	80 (3.2)		1868 (75.4)	149 (6.0)	74 (3.0)
7–13										
2010	2 327	244 (10.5)	178 (7.6)		137 (5.9)	130 (5.6)		1501 (64.5)	80 (3.4)	57 (2.4)
2012	2 973	278 (9.4)	117 (3.9)		224 (7.5)	227 (7.6)		1914 (64.4)	148 (5.0)	65 (2.2)
2014	3 026	237 (7.8)	114 (3.8)		220 (7.3)	125 (4.1)		2068 (68.3)	184 (6.1)	78 (2.6)
2017	2 807	181 (6.4)	109 (3.9)		128 (4.6)	50 (1.8)		1984 (70.7)	227 (8.1)	128 (4.6)
14–18										
2010	1 761	237 (13.5)	128 (7.3)		105 (6.0)	68 (3.9)		1086 (61.7)	92 (5.2)	45 (2.6)
2012	1 803	219 (12.1)	102 (5.7)		157 (8.7)	90 (5.0)		1043 (57.8)	113 (6.3)	79 (4.4)
2014	1 953	202 (10.3)	188 (9.6)		133 (6.8)	63 (3.2)		1095 (56.1)	147 (7.5)	125 (6.4)
2017	1 803	162 (9.0)	112 (6.2)		76 (4.2)	46 (2.6)		984 (54.6)	194 (10.8)	229 (12.7)
19–24										
2010	1 455	187 (12.9)	139 (9.6)		100 (6.9)	47 (3.2)		751 (51.6)	120 (8.2)	111 (7.6)
2012	1 563	209 (13.4)	144 (9.2)		102 (6.5)	78 (5.0)		713 (45.6)	140 (9.0)	177 (11.3)
2014	1 703	190 (11.2)	171 (10.0)		107 (6.3)	63 (3.7)		708 (41.6)	201 (11.8)	263 (15.4)
2017	1 581	118 (7.5)	97 (6.1)		62 (3.9)	50 (3.2)		648 (41.0)	254 (16.1)	352 (22.3)
25–34										
2010	1 597	186 (11.6)	175 (11.0)		97 (6.1)	90 (5.6)		590 (36.9)	188 (11.8)	271 (17.0)
2012	1 721	199 (11.6)	165 (9.6)		117 (6.8)	113 (6.6)		583 (33.9)	222 (12.9)	322 (18.7)
2014	1 831	148 (8.1)	167 (9.1)		116 (6.3)	75 (4.1)		612 (33.4)	256 (14.0)	457 (25.0)
2017	1 705	84 (4.9)	85 (5.0)		80 (4.7)	70 (4.1)		598 (35.1)	282 (16.5)	506 (29.7)
35–44										
2010	1 514	121 (8.0)	147 (9.7)		84 (5.5)	112 (7.4)		423 (27.9)	186 (12.3)	441 (29.1)
2012	1 634	110 (6.7)	156 (9.5)		89 (5.4)	123 (7.5)		439 (26.9)	226 (13.8)	491 (30.0)
2014	1 647	91 (5.5)	141 (8.6)		82 (5.0)	80 (4.9)		458 (27.8)	225 (13.7)	570 (34.6)
2017	1 556	65 (4.2)	64 (4.1)		60 (3.9)	77 (4.9)		438 (28.1)	246 (15.8)	606 (38.9)
45–54										
2010	1 305	92 (7.0)	130 (10.0)		64 (4.9)	96 (7.4)		334 (25.6)	168 (12.9)	421 (32.3)
2012	1 415	91 (6.4)	111 (7.8)		67 (4.7)	130 (9.2)		355 (25.1)	193 (13.6)	468 (33.1)
2014	1 370	68 (5.0)	113 (8.2)		66 (4.8)	78 (5.7)		343 (25.0)	213 (15.5)	489 (35.7)
2017	1 260	32 (2.5)	48 (3.8)		53 (4.2)	64 (5.1)		344 (27.3)	230 (18.3)	489 (38.8)
≥ 55										
2010	1 712	118 (6.9)	170 (9.9)		111 (6.5)	140 (8.2)		462 (27.0)	248 (14.5)	463 (27.0)
2012	1 754	132 (7.5)	134 (7.6)		123 (7.0)	197 (11.2)		442 (25.2)	265 (15.1)	461 (26.3)
2014	1 522	85 (5.6)	117 (7.7)		109 (7.2)	98 (6.4)		414 (27.2)	257 (16.9)	442 (29.0)
2017	1 272	37 (2.9)	46 (3.6)		76 (6.0)	91 (7.2)		390 (30.7)	227 (17.8)	405 (31.8)
**Race**										
African										
2010	11 536	1192 (10.3)	1134 (9.8)		777 (6.7)	764 (6.6)		5129 (44.5)	969 (8.4)	1571 (13.6)
2012	12 941	1225 (9.5)	981 (7.6)		1033 (8.0)	1130 (8.7)		5709 (44.1)	1152 (8.9)	1711 (13.2)
2014	13 228	1021 (7.7)	950 (7.2)		971 (7.3)	746 (5.6)		6234 (47.1)	1323 (10.0)	1983 (15.0)
2017	12 317	696 (5.7)	531 (4.3)		583 (4.7)	451 (3.7)		6251 (50.8)	1521 (12.3)	2284 (18.5)
Mixed ancestry										
2010	1 400	86 (6.1)	78 (5.6)		88 (6.3)	71 (5.1)		696 (49.7)	134 (9.6)	247 (17.6)
2012	1 887	130 (6.9)	116 (6.1)		77 (4.1)	107 (5.7)		975 (51.7)	158 (8.4)	324 (17.2)
2014	1 923	109 (5.7)	122 (6.3)		70 (3.6)	56 (2.9)		1001 (52.1)	183 (9.5)	382 (19.9)
2017	1 770	91 (5.1)	76 (4.3)		48 (2.7)	63 (3.6)		892 (50.4)	203 (11.5)	397 (22.4)
Asian										
2010	128	10 (7.8)	5 (3.9)		5 (3.9)	4 (3.1)		66 (51.6)	18 (14.1)	20 (15.6)
2012	141	13 (9.2)	8 (5.7)		7 (5.0)	5 (3.5)		58 (41.1)	28 (19.9)	22 (15.6)
2014	152	13 (8.6)	9 (5.9)		11 (7.2)	7 (4.6)		65 (42.8)	23 (15.1)	24 (15.8)
2017	145	11 (7.6)	7 (4.8)		7 (4.8)	6 (4.1)		56 (38.6)	32 (22.1)	26 (17.9)
Caucasian										
2010	234	25 (10.7)	18 (7.7)		17 (7.3)	21 (9.0)		62 (26.5)	42 (17.9)	49 (20.9)
2012	362	35 (9.7)	47 (13.0)		16 (4.4)	25 (6.9)		80 (22.1)	75 (20.7)	84 (23.2)
2014	320	20 (6.3)	22 (6.9)		24 (7.5)	20 (6.3)		66 (20.6)	65 (20.3)	103 (32.2)
2017	231	11 (4.8)	9 (3.9)		13 (5.6)	8 (3.5)		55 (23.8)	53 (22.9)	82 (35.5)
**Residence**										
Rural										
2010	7 937	882 (11.1)	826 (10.4)		536 (6.8)	508 (6.4)		3585 (45.2)	654 (8.2)	946 (11.9)
2012	8 420	801 (9.5)	625 (7.4)		711 (8.4)	793 (9.4)		3780 (44.9)	743 (8.8)	967 (11.5)
2014	8 152	609 (7.5)	572 (7.0)		639 (7.8)	515 (6.3)		3920 (48.1)	788 (9.7)	1109 (13.6)
2017	7 501	410 (5.5)	289 (3.9)		365 (4.9)	283 (3.8)		3929 (52.4)	928 (12.4)	1297 (17.3)
Urban										
2010	5 361	431 (8.0)	409 (7.6)		351 (6.5)	352 (6.6)		2368 (44.2)	509 (9.5)	941 (17.6)
2012	6 911	602 (8.7)	527 (7.6)		422 (6.1)	474 (6.9)		3042 (44.0)	670 (9.7)	1174 (17.0)
2014	7 471	554 (7.4)	531 (7.1)		437 (5.8)	314 (4.2)		3446 (46.1)	806 (10.8)	1383 (18.5)
2017	6 962	399 (5.7)	334 (4.8)		286 (4.1)	245 (3.5)		3325 (47.8)	881 (12.7)	1492 (21.4)
**Education**										
None										
2010	1 391	115 (8.3)	145 (10.4)		84 (6.0)	104 (7.5)		506 (36.4)	153 (11.0)	284 (20.4)
2012	1 437	134 (9.3)	105 (7.3)		102 (7.1)	156 (10.9)		519 (36.1)	160 (11.1)	261 (18.2)
2014	1 345	90 (6.7)	85 (6.3)		107 (8.0)	76 (5.7)		543 (40.4)	172 (12.8)	272 (20.2)
2017	1 168	52 (4.5)	36 (3.1)		65 (5.6)	53 (4.5)		506 (43.3)	181 (15.5)	275 (23.5)
Pre-school										
2010	1 508	116 (7.7)	160 (10.6)		181 (12.0)	165 (10.9)		735 (48.7)	74 (4.9)	77 (5.1)
2012	2 324	153 (6.6)	212 (9.1)		245 (10.5)	295 (12.7)		1247 (53.7)	95 (4.1)	77 (3.3)
2014	2 418	127 (5.3)	87 (3.6)		224 (9.3)	238 (9.8)		1571 (65.0)	104 (4.3)	67 (2.8)
2017	2 338	118 (5.0)	59 (2.5)		106 (4.5)	78 (3.3)		1768 (75.6)	139 (5.9)	70 (3.0)
Primary school^a^										
2010	8 441	868 (10.3)	718 (8.5)		505 (6.0)	477 (5.7)		4041 (47.9)	692 (8.2)	1140 (13.5)
2012	9 335	865 (9.3)	617 (6.6)		657 (7.0)	671 (7.2)		4389 (47.0)	849 (9.1)	1287 (13.8)
2014	9 573	753 (7.9)	685 (7.2)		631 (6.6)	415 (4.3)		4608 (48.1)	962 (10.0)	1519 (15.9)
2017	8 864	518 (5.8)	402 (4.5)		405 (4.6)	317 (3.6)		4391 (49.5)	1101 (12.4)	1730 (19.5)
High school and above										
2010	1 958	214 (10.9)	212 (10.8)		117 (6.0)	114 (5.8)		671 (34.3)	244 (12.5)	386 (19.7)
2012	2 235	251 (11.2)	218 (9.8)		129 (5.8)	145 (6.5)		667 (29.8)	309 (13.8)	516 (23.1)
2014	2 287	193 (8.4)	246 (10.8)		114 (5.0)	100 (4.4)		644 (28.2)	356 (15.6)	634 (27.7)
2017	2 093	121 (5.8)	126 (6.0)		75 (3.6)	80 (3.8)		589 (28.1)	388 (18.5)	714 (34.1)
**Income level**										
Low										
2010	8 162	897 (11.0)	803 (9.8)		555 (6.8)	529 (6.5)		3798 (46.5)	617 (7.6)	963 (11.8)
2012	9 264	894 (9.7)	647 (7.0)		779 (8.4)	811 (8.8)		4330 (46.7)	753 (8.1)	1050 (11.3)
2014	9 582	733 (7.6)	644 (6.7)		747 (7.8)	541 (5.6)		4827 (50.4)	871 (9.1)	1219 (12.7)
2017	8 974	525 (5.9)	373 (4.2)		418 (4.7)	298 (3.3)		4876 (54.3)	1024 (11.4)	1460 (16.3)
Middle										
2010	3 243	276 (8.5)	254 (7.8)		216 (6.7)	187 (5.8)		1486 (45.8)	295 (9.1)	529 (16.3)
2012	3 780	294 (7.8)	297 (7.9)		238 (6.3)	289 (7.6)		1734 (45.9)	348 (9.2)	580 (15.3)
2014	3 776	271 (7.2)	266 (7.0)		216 (5.7)	174 (4.6)		1769 (46.8)	387 (10.2)	693 (18.4)
2017	3 501	193 (5.5)	140 (4.0)		154 (4.4)	148 (4.2)		1673 (47.8)	443 (12.7)	750 (21.4)
High										
2010	1 893	140 (7.4)	178 (9.4)		116 (6.1)	144 (7.6)		669 (35.3)	251 (13.3)	395 (20.9)
2012	2 287	215 (9.4)	208 (9.1)		116 (5.1)	167 (7.3)		758 (33.1)	312 (13.6)	511 (22.3)
2014	2 265	159 (7.0)	193 (8.5)		113 (5.0)	114 (5.0)		770 (34.0)	336 (14.8)	580 (25.6)
2017	1 988	91 (4.6)	110 (5.5)		79 (4.0)	82 (4.1)		705 (35.5)	342 (17.2)	579 (29.1)
**Physical exercise**										
< 3 times per week										
2010	7 950	769 (9.7)	792 (10.0)		487 (6.1)	497 (6.3)		2868 (36.1)	902 (11.3)	1635 (20.6)
2012	8 315	799 (9.6)	724 (8.7)		530 (6.4)	648 (7.8)		2754 (33.1)	1011 (12.2)	1849 (22.2)
2014	8 444	636 (7.5)	784 (9.3)		502 (5.9)	412 (4.9)		2823 (33.4)	1135 (13.4)	2152 (25.5)
2017	7 742	399 (5.2)	387 (5.0)		354 (4.6)	349 (4.5)		2653 (34.3)	1236 (16.0)	2364 (30.5)
≥ 3 times per week										
2010	985	125 (12.7)	64 (6.5)		51 (5.2)	40 (4.1)		522 (53.0)	81 (8.2)	102 (10.4)
2012	1 135	119 (10.5)	73 (6.4)		85 (7.5)	57 (5.0)		546 (48.1)	127 (11.2)	128 (11.3)
2014	1 143	102 (8.9)	87 (7.6)		75 (6.6)	35 (3.1)		536 (46.9)	141 (12.3)	167 (14.6)
2017	1 040	64 (6.2)	47 (4.5)		38 (3.7)	38 (3.7)		505 (48.6)	160 (15.4)	188 (18.1)
Unknown										
2010	4 363	419 (9.6)	379 (8.7)		349 (8.0)	323 (7.4)		2563 (58.7)	180 (4.1)	150 (3.4)
2012	5 881	485 (8.2)	355 (6.0)		518 (8.8)	562 (9.6)		3522 (59.9)	275 (4.7)	164 (2.8)
2014	6 036	425 (7.0)	232 (3.8)		499 (8.3)	382 (6.3)		4007 (66.4)	318 (5.3)	173 (2.9)
2017	5 681	346 (6.1)	189 (3.3)		259 (4.6)	141 (2.5)		4096 (72.1)	413 (7.3)	237 (4.2)
**Total**										
2010	13 298	1313 (9.9)	1235 (9.3)		887 (6.7)	860 (6.5)		5953 (44.8)	1163 (8.7)	1887 (14.2)
2012	15 331	1403 (9.2)	1152 (7.5)		1133 (7.4)	1267 (8.3)		6822 (44.5)	1413 (9.2)	2141 (14.0)
2014	15 623	1163 (7.4)	1103 (7.1)		1076 (6.9)	829 (5.3)		7366 (47.1)	1594 (10.2)	2492 (16.0)
2017	14 463	809 (5.6)	623 (4.3)		651 (4.5)	528 (3.7)		7254 (50.2)	1809 (12.5)	2789 (19.3)

^a^ Includes those who may have started high school but dropped out before completing.

### Likelihood of transitions

The probability of transitioning upwards or downwards to another BMI category decreased with time, while the probability of remaining within the overweight or obese categories increased with time. The particular groups that demonstrated the largest probabilities of remaining within the overweight or obese categories, compared with other groups, also demonstrated the greatest likelihoods of transitioning either upwards or downwards. These groups included women, Caucasians, and those with at least a high school education and with a high income (Table 4). 

**Table 4:** Probability of transitioning from one body mass index category to another (or not) by sociodemographic characteristics and year of end of transition period, South Africa, 2010–2017

Sociodemographic factor/year of end of transition period	OR (*P*) relative to that of retaining a normal BMI		
	Transition upwards^a^	Transition downwards^b^	Remain obese or overweight
**End of transition period **			
2010	1.00	1.00	1.00
2012	0.93 (0.032)	1.24 (0.000)	1.13 (0.000)
2014	0.77 (0.000)	0.93 (0.066)	1.28 (0.000)
2017	0.50 (0.000)	0.59 (0.000)	1.56 (0.000)
**Sex **			
Male	1.00	1.00	1.00
Female	2.56 (0.000)	1.82 (0.000)	4.81 (0.000)
**Baseline age (years)**			
0–6	1.00	1.00	1.00
7–13	0.93 (0.565)	0.72 (0.009)	1.14 (0.435)
14–18	1.21 (0.196)	0.71 (0.021)	1.50 (0.028)
19–24	1.40 (0.029)	0.81 (0.180)	2.75 (0.000)
25–34	1.59 (0.003)	1.19 (0.276)	4.92 (0.000)
35–44	1.65 (0.001)	1.54 (0.007)	8.33 (0.000)
45–54	1.67 (0.001)	1.75 (0.001)	10.44 (0.000)
≥ 55	1.65 (0.001)	2.17 (0.000)	9.68 (0.000)
**Race **			
African	1.00	1.00	1.00
Mixed ancestry	0.64 (0.000)	0.54 (0.000)	0.68 (0.000)
Asian	0.68 (0.005)	0.60 (0.001)	0.71 (0.003)
Caucasian	1.39 (0.002)	1.26 (0.043)	1.21 (0.037)
**Residence **			
Rural	1.00	1.00	1.00
Urban	0.85 (0.000)	0.92 (0.005)	1.08 (0.002)
**Education level **			
None	1.00	1.00	1.00
Pre-school	1.11 (0.448)	1.50 (0.003)	1.76 (0.001)
Primary school^c^	1.16 (0.005)	1.18 (0.002)	1.50 (0.000)
High school and above	1.69 (0.000)	1.43 (0.000)	2.27 (0.000)
**Income level**			
Low	1.00	1.00	1.00
Middle	0.98 (0.623)	0.94 (0.066)	1.18 (0.000)
High	1.46 (0.000)	1.28 (0.000)	2.06 (0.000)
**Physical exercise **			
< 3 times per week	1.00	1.00	1.00
≥ 3 times per week	0.93 (0.160)	0.86 (0.014)	0.83 (0.000)
Unknown	0.77 (0.001)	0.91 (0.322)	0.66 (0.000)

BMI: body mass index; OR: odds ratio.

^a^ Transition from normal to overweight, or overweight to obese.

^b^ Transition from obese to overweight, or overweight to normal.

^c^ Includes those who may have started high school but dropped out before completing.

We note that the likelihood of either transitioning upwards or downwards, or of remaining within the overweight or obese categories, relative to that of retaining a normal BMI, increased with age. Our data show that women were 2.56 and 1.82 times more likely than men to transition upwards and downwards, respectively, and 4.81 times more likely than men to remain within the overweight or obese category. Men were therefore more likely to retain a normal BMI. 

We note that Caucasians were 1.39 and 1.26 times more likely than Africans to transition upwards and downwards, respectively, and 1.21 times more likely than Africans to remain within the overweight or obesity category. Those of mixed ancestry and Asians demonstrated a higher probability of retaining a normal BMI than Africans and Caucasians. Urban dwellers were slightly more likely (1.08 times) than rural dwellers to remain within the overweight or obese categories, but less likely to transition upwards or downwards. We observed that individuals with a high school education and greater were more likely than the other education-level groups to either transition upwards or downwards or to remain within the overweight or obese categories. Compared with those with a low or middle income, high-income groups were more likely to transition upwards or downwards and remain within the overweight or obese categories; high-income groups were therefore less likely to retain a normal BMI than lower-income groups. Those who exercised ≥ 3 times per week and those whose frequency of physical exercise was unknown (data were unavailable for those of age < 15 years) were less likely to remain within the overweight or obese categories, and therefore more likely to retain a normal BMI, compared with those who exercised < 3 times per week. 

## Discussion

Our data show a sharply rising prevalence of obesity coinciding with entry into adulthood in the community at large. We note that the prevalence of obesity increased by the greatest amount for the group aged 19–24 years between 2008 and 2017. This age group also reported the highest percentage of upwards transitions, as well as the largest difference between the percentage transitioning upwards and the percentage transitioning downwards. Although this age group had a normal-category mean BMI during the first two study periods, the subsequent sharp increase in BMI resulted in an increased prevalence of being overweight and obese with time. 

The South African Department of Health targeted a 3% reduction in the prevalence of obesity by 2017 for all age groups through diet and physical activity.^8^^,^^9^ To determine whether this target was achieved, we must compare data from different individuals of the same age at different times. However, by examining how BMI values change as a particular cohort ages, we were able to identify periods of increased obesity prevalence within a person’s lifetime during which interventions may be critical. Our data show that there exists a decreasing trend in prevalence of being overweight or obese from birth to the age of 18 years, when most children usually leave high school. As our results are not capable of attributing this downwards trajectory from birth to age 18 years to the effectiveness of national strategies for obesity prevention and control (our data were obtained from an observational study), further research is required. 

Guidance for healthy eating for the population aged ≤ 18 years is provided in South Africa by the *Guidelines for early childhood development services*,^15^^,^^16^ and the *National school nutrition programme annual report*^17^ and *Guidelines for tuck shop operators*.^18^ The teaching of life skills in primary and high schools, which includes nutrition education, is aimed at educating students to make nutritious food choices and develop healthy eating habits.^9^ In averaging our data for all ages over the period 2008–2017, we found that the lowest combined prevalence of being overweight and obese was for those aged 7–18 years; the fact that prevalence rises steadily after this age highlights that, despite these strategies on controlling obesity, there remain substantial problems in communicating healthy food choices to children and adolescents. Other studies have also shown that these strategies are not functioning optimally.^19^^–^^21^ A synthesis of studies conducted between 2006 and 2014 on the South African school food environment revealed that over half (from 51.1%; 1233/2412 to 69.3%; 330/476) of the students bought available and unhealthy foods from either tuck shops or vendors in their neighbourhood.^22^ The population of age 15–24 years has also been identified as the largest consumers of sugar-sweetened beverages.^23^

Low levels of physical activity, especially in female adolescents (aged 10–19 years)^24^ and in older teenagers (aged 16–18 years) of both sexes, could also be contributing to the existing prevalence of being overweight and obese.^25^ Physical activity policies include the *Strategic plan 2009–2013: An active and winning nation*,^26^ of which one of the aims was to facilitate the implementation of sports in schools. In the *National sport and recreation plan*, one of the objectives is to maximize access to sport, recreation and physical education in every school.^27^ Consolidated findings on the level of physical activity in children aged 3–19 years (e.g. early childhood physical activity, organized sport participation, active play and active transportation) showed an average participation of around 50%, although the percentage of those in early childhood (age 3–6 years) participating in physical activity was generally found to be high (~80%).^25^ Since pre-school children are naturally active, a physical activity intervention for this age group was only deemed necessary for promoting the engagement of teachers and parents or caregivers, and for outcomes such as cognitive development.^28^

The South African noncommunicable disease strategy^8^ targeted an increase in the prevalence of physical activity (150 minutes of moderate-intensity physical activity per week, or equivalent) by 10% by 2020, and the WHO global action plan for noncommunicable diseases^5^ targeted a 10% relative reduction in the prevalence of insufficient physical activity by 2020. Although our data do not allow a detailed analysis of types of physical activity, we demonstrated the importance of physical exercise. About half of those who exercised ≥ 3 times per week maintained a normal BMI even as the cohort aged. However, the fact that the proportion of this group who maintained a normal BMI decreased slightly over time may reflect decreasing levels of energy expenditure with age. Interventions that increase physical activity may therefore be necessary to reduce the large percentages of groups who remain either overweight or obese. Studies on obesity, especially in women, have identified obese participants who meet the WHO guidelines on physical activity but have very poor cardiorespiratory fitness, possibly attributable to a lack of high-intensity physical activity.^29^^,^^30^ Suggested interventions include encouraging participation in high-intensity activities, such as sports and aerobics, and discouraging sedentary behaviour.^31^

The recent introduction of higher tax on products such as sugar-sweetened beverages^32^ will probably reduce consumption and consequently reduce the percentages transitioning upwards. However, the life course approach, the basis of many WHO strategies and recommendations for disease prevention and control,^5^^,^^33^ may be a better strategy for maintaining normal BMI from this age. The life course approach stresses the importance of early intervention by considering which stages, transitions and settings of a person’s life are critical for promoting or restoring health.^34^ For example, the workplace has been found to be an important setting for health promotion during adulthood.^35^ Effective interventions include the promotion of healthy food options in canteens, and the provision of nutrition education and counselling.^36^ Similar interventions in settings such as community centres, churches and recreational facilities should also be promoted.^37^ Barriers to leisure activity participation, including lack of time and facilities, safety issues and negative community perceptions regarding weight loss, may also need to be addressed,^38^ along with the accessibility and affordability of healthy food options through subsidies.^37^

Population groups that demonstrated a higher probability of remaining within the obese category included women, higher age groups (> 35 years), and those with a higher income and higher level of education. These groups are usually the target of public interventions and of the weight management programmes of private health-care providers.^39^ However, our data show a high resistance to a downwards transition from either overweight or obese categories, and this resistance increases with time. Other studies have shown that although weight loss can be achieved through lifestyle changes,^40^^,^^41^ maintaining this weight loss over the longer term can be difficult; only approximately one-fifth (20.6%; 47/228)^42^ of those who achieve weight loss maintain it for ≥ 1 year,^42^^,^^43^ and the majority of those who embark on a weight-loss programme give up before achieving success.^44^^–^^47^ Our results indicate that, despite considerable percentages of the cohort transitioning downwards, any reductions in the prevalence of obesity were cancelled out by the larger percentages of these population groups transitioning upwards; we therefore believe that the South African target^9^ of achieving a 10% reduction in the prevalence of obesity by 2020 will not be met. 

The main strength of our study was our analysis of BMI transitions within a nationally representative sample of participants from a panel survey spanning birth to adulthood. However, our study had limitations. Some of the risk factors associated with higher BMI categories in this study, such as household income, education level and amount of physical exercise, were self-reported and therefore prone to minor errors that may have affected our results. A second limitation was loss to follow-up from one wave of data collection to another. Since we assume those lost to follow-up were randomly distributed throughout the study population, and the remaining numbers at each subsequent data collection wave were sufficiently high, we consider our results to be free of bias.

To conclude, we have demonstrated that the proportion of upwards transitions to overweight and obese categories in the South African population increases sharply between the ages of 19 and 50 years. Once overweight or obese, the likelihood of transitioning to a normal BMI is low, particularly for women, those of higher age groups, and those with a higher income and a higher level of education. With this evidence, we provide essential guidance for the South African Department of Health in the development of strategies to control obesity and noncommunicable diseases. Our study results will allow limited public health resources to be focused on the necessary population segments in which the largest reductions in the prevalence of obesity can be made.
